# Age-Stratified Transcriptomic Profiling Reveals Biologically Distinct Molecular Phenotypes Across Pediatric, Adolescent, and Adult Osteosarcoma

**DOI:** 10.3390/biomedicines14020363

**Published:** 2026-02-04

**Authors:** Li Hu, Feiyang Qi, Huimin Liu, Yiping Cao, Qinghua Li, Haijie Liang, Xingyu Liu, Zhiye Du, Yang Wang, Jichuan Wang

**Affiliations:** 1 Familial & Hereditary Cancer Center, Key Laboratory of Carcinogenesis and Translational Research (Ministry of Education), Peking University Cancer Hospital & Institute, Beijing 100142, China; 2165610821@bjmu.edu.cn; 2 Comprehensive Clinical Trial Ward, Key Laboratory of Carcinogenesis and Translational Research (Ministry of Education), Peking University Cancer Hospital & Institute, Beijing 100142, China; 3 Musculoskeletal Tumor Center, Beijing Key Laboratory for Musculoskeletal Tumors, Peking University People’s Hospital, Beijing 100044, China; 1910301241@pku.edu.cn (F.Q.); lianghaijie_glia@163.com (H.L.); lxysunknight@bjmu.edu.cn (X.L.); duzhiye0527@gmail.com (Z.D.); 4 Multidisciplinary Diagnosis and Treatment Center for Bone Tumors, Peking University Shougang Hospital, Beijing 100144, China; liuhuimin0122@163.com; 5 Department of Biochemistry and Molecular Biology, School of Basic Medical Sciences, Peking University International Cancer Institute, Peking University Health Science Center, Beijing 100191, China; yipingcao@hsc.pku.edu.cn (Y.C.); 2211110050@bjmu.edu.cn (Q.L.)

**Keywords:** pediatric osteosarcoma, transcriptomics, age stratification, prognostic signature, tumor microenvironment, cell proliferation, risk stratification

## Abstract

**Background/Objectives**: Osteosarcoma exhibits bimodal age distribution with distinct clinical behaviors between pediatric and adult patients. Despite genomic evidence supporting age-related molecular heterogeneity, systematic transcriptomic characterization remains lacking. This study aimed to delineate age-associated transcriptional differences and develop a pediatric-specific prognostic signature. **Methods**: Bulk RNA sequencing was performed on tumor specimens from 70 osteosarcoma patients stratified into pediatric (≤14 years, *n* = 37), adolescent (15–18 years, *n* = 22), and adult (≥19 years, *n* = 11) groups. Differential expression, functional enrichment, and immune infiltration analyses were conducted. A pediatric-specific signature was validated in the TARGET-OS cohort (*n* = 87). **Results**: Pediatric osteosarcoma exhibited a hyperproliferative phenotype, enriched in E2F targets, G2M checkpoint, and DNA replication pathways. Adolescent tumors showed heightened immune–inflammatory signatures, while adult tumors activated osteogenic differentiation programs. Regarding the immune microenvironment, only adolescent tumors demonstrated active immune infiltration; pediatric and adult groups exhibited immunologically “cold” features. We identified a 10-gene pediatric-specific transcriptomic signature that declined with increasing age. High signature scores were significantly associated with inferior overall survival (hazard ratio [HR] = 5.6, 95% confidence interval [CI]: 1.2–26.2, *p* = 0.01) and progression-free survival (HR = 2.1, 95% CI: 1.1–4.2, *p* = 0.03). These findings showed concordant trends in the independent TARGET-OS cohort. **Conclusions**: Pediatric, adolescent, and adult osteosarcoma harbor distinct transcriptional profiles representing biologically different disease entities. The pediatric-specific 10-gene signature may serve as a clinically actionable biomarker for risk stratification and guide age-adapted therapeutic strategies.

## 1. Introduction

Osteosarcoma (OS) is the most common primary malignant bone tumor, accounting for approximately 35% of all bone malignancies [1]. The disease exhibits a characteristic bimodal age distribution, with an initial peak during adolescence (10–19 years) coinciding with the pubertal growth spurt, and a secondary peak in adults over 60 years of age [1,2]. These two populations differ not only in epidemiological patterns but also in clinical presentation: pediatric OS typically arises in the metaphyses of long bones during periods of rapid skeletal growth [3], whereas adult-onset cases are more frequently associated with secondary etiologies and may demonstrate distinct metastatic behaviors and treatment responses [4]. Despite these recognized clinical differences, current therapeutic approaches remain largely uniform across age groups, relying on neoadjuvant chemotherapy followed by surgical resection. While this standardized regimen achieves approximately 70% five-year survival rates for patients with localized disease, outcomes for those with metastatic or recurrent OS remain poor, with survival rates below 20% [5,6]. These outcomes have not substantially improved over the past four decades, highlighting the need for more refined, biologically informed treatment strategies.

Genomic studies have begun to characterize the molecular landscape of OS across different age groups. Unlike many solid tumors that harbor recurrent driver mutations, OS is defined by extensive chromosomal instability, including chromothripsis, kataegis, and widespread copy number alterations (CNAs) [7,8,9,10]. Within this complex genomic background, age-associated differences have been identified. Zou and colleagues analyzed 194 patients and found that homologous recombination deficiency (HRD)-related alterations were approximately twice as frequent in younger patients compared to older adults (~17% vs. ~8%) [11,12]. Large-scale clinical sequencing has further confirmed these patterns, with PDGFRA and VEGFA amplifications predominating in younger cohorts and CDK4/MDM2 amplifications more common in older patients [13]. However, the functional and clinical implications of these genomic differences remain incompletely understood, and genomic data alone have not yet translated into age-specific therapeutic strategies.

Transcriptomic profiling offers a complementary approach to understanding OS biology, as it captures the functional state of gene expression and tumor-microenvironment interactions. Recent advances in RNA sequencing have enabled detailed characterization of the OS immune landscape [14,15,16,17] and identification of molecular subtypes with differential chemotherapy sensitivity [18]. However, these studies have primarily analyzed mixed-age cohorts without systematic stratification by age group. As a result, whether pediatric and adult OS exhibit distinct transcriptomic signatures, and whether such differences carry prognostic significance, remains unclear. This gap in knowledge limits the development of age-appropriate risk stratification models and targeted therapeutic approaches.

In this study, we conducted comprehensive bulk RNA sequencing on tumor samples derived from 70 individuals diagnosed with osteosarcoma, meticulously categorized into three cohorts: pediatric patients (≤14 years), adolescents (15–18 years), and adults (≥19 years). We comprehensively compared transcriptomic differences across age groups and explored their potential clinical implications, with findings independently validated in the TARGET-OS cohort. These results will contribute to a comprehensive transcriptomic framework delineating age-dependent molecular heterogeneity in osteosarcoma and provide a clinically applicable tool for risk stratification.

## 2. Materials and Methods

### 2.1. Patient Cohort and Sample Collection

**Figure 1:**
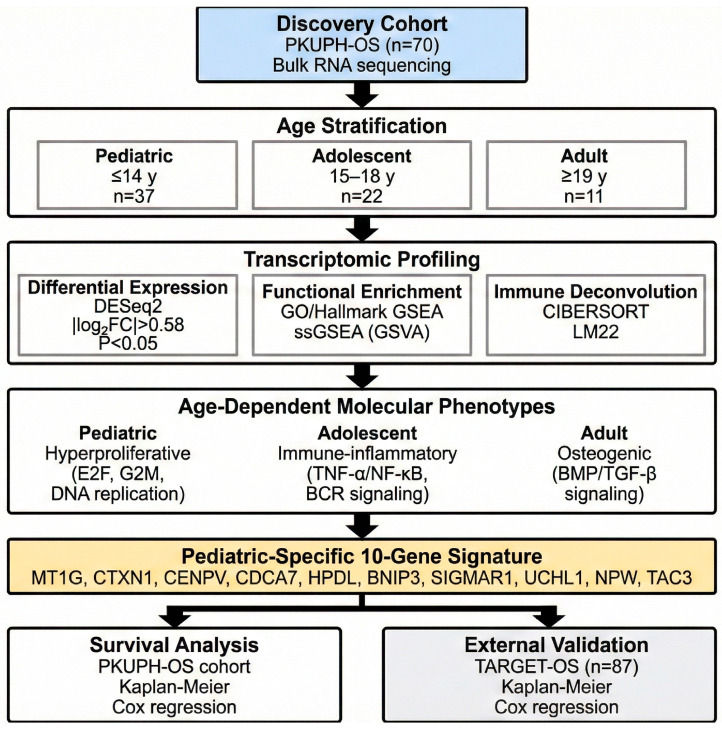
Schematic overview of the study design. Overview of the study workflow, including patient cohort stratification into pediatric (≤14 years), adolescent (15–18 years), and adult (≥19 years) groups. The diagram illustrates the integrated pipeline of bulk RNA sequencing, differential expression analysis, immune microenvironment characterization, and the development and validation of the pediatric-specific 10-gene prognostic signature.

Our retrospective cohort comprised 70 osteosarcoma patients treated at Peking University People’s Hospital from June 2012 to December 2023. To ensure data integrity, we only included cases with histologically confirmed primary disease and characteristic radiographic features. All subjects had undergone complete surgical resection and were followed for at least three months, with any patients presenting concurrent malignancies being excluded. We extracted comprehensive clinicopathological and therapeutic data from electronic medical records, a process double-checked by two independent investigators; a third senior reviewer was consulted to settle any conflicting data points. Following a standardized treatment protocol as previously defined, patients were categorized into three developmental stages at the time of diagnosis: pediatric (≤14 years), adolescent (15–18 years), and adult (≥19 years). The age-based stratification in this study was defined to reflect both the biological growth phases and clinical oncology standards. The cutoff of ≤14 years corresponds to the peak incidence observed in late childhood/early puberty as reported in SEER data. The 15–18 years range captures the window of maximal pubertal growth spurt associated with osteosarcoma pathogenesis, while patients ≥19 years represent the transition to adult clinical management protocols, a division consistent with previous large-scale cohort studies [19,20]. The overall experimental design and analytical workflow of this study are summarized in Figure 1. This study adhered to the Declaration of Helsinki and received formal clearance from the Ethics Committee of Peking University People’s Hospital (No. 2024PHB432-001), with written informed consent obtained from all participants.

### 2.2. External Validation Cohort

For external validation, transcriptomic data and corresponding clinical information from 87 patients with primary osteosarcoma were obtained from the Therapeutically Applicable Research to Generate Effective Treatments (TARGET) Osteosarcoma Project (TARGET-OS; https://ocg.cancer.gov/programs/target, accessed on 23 October 2024). Gene expression profiles were processed and normalized as described in the original dataset publications.

### 2.3. Bulk RNA-Seq and Data Analysis

Fresh-frozen tumor specimens from 70 patients were processed under sterile conditions, immediately cryopreserved in liquid nitrogen, and maintained at −80 °C until analysis. Total RNA isolation was performed using TRIzol reagent (Invitrogen, Carlsbad, CA, USA) in accordance with the manufacturer’s guidelines. RNA purity and concentration were determined using a NanoDrop 2000 spectrophotometer (Thermo Fisher Scientific, Waltham, MA, USA), and RNA integrity was evaluated using an Agilent 2100 Bioanalyzer (Agilent Technologies, Santa Clara, CA, USA). Samples with RNA integrity number (RIN) ≥ 7 were subjected to library construction. Sequencing libraries were prepared using the VAHTS Universal V6 RNA-seq Library Prep Kit (Vazyme, Nanjing, China) according to the manufacturer’s instructions, and sequenced on an Illumina NovaSeq 6000 platform, generating 150-bp paired-end reads. Following quality filtering and adapter trimming, raw sequencing reads were aligned to the human reference genome (hg38) using STAR (v2.7) [21], after which gene-level expression was quantified using featureCounts [22] and subsequently normalized as transcripts per million (TPM) for downstream analyses.

### 2.4. Differential Gene Expression Analysis

Pairwise differential gene expression analyses were performed among the three age groups (pediatric patients vs. adults, pediatric patients vs. adolescents, and adolescents vs. adults) using the DESeq2 package [23] in R (v4.2.0). Prior to differential expression analysis, raw count data were normalized using the variance stabilizing transformation (VST) method. Genes meeting the thresholds of *p* value < 0.05 (Benjamini–Hochberg correction) and |log_2_(fold change)| > 0.58 were defined as significantly differentially expressed genes (DEGs), and their distribution was subsequently visualized using volcano plots generated with the EnhancedVolcano package (v1.16.0).

### 2.5. Unsupervised Clustering Analysis

To explore transcriptomic patterns associated with age, unsupervised hierarchical clustering was performed on the expression profiles of age-associated DEGs using the ComplexHeatmap package [24] in R. Euclidean distance served as the distance metric, with Ward’s method (Ward.D2) applied for linkage. Prior to clustering and visualization, expression values were scaled across samples using z-score normalization to ensure comparability.

### 2.6. Functional Enrichment Analysis

Gene Set Enrichment Analysis (GSEA) was performed using the clusterProfiler package [25] in R to identify biological processes and pathways associated with age-related transcriptomic differences. GSEA was conducted with Gene Ontology (GO) gene sets to assess enrichment in biological processes, molecular functions, and cellular components. Additionally, GSEA was performed with the Hallmark gene sets (MSigDB, v7.5.1) [26] to evaluate pathway-level enrichment. Pathways with an adjusted *p* value < 0.05 and |normalized enrichment score (NES)| > 1 were considered significantly enriched.

### 2.7. Single-Sample Gene Set Enrichment Analysis (ssGSEA)

Single-sample GSEA (ssGSEA) was performed using the GSVA package [27] in R to quantify the enrichment of predefined gene sets at the individual sample level. Gene sets related to cell cycle progression (such as E2F targets and G2M checkpoint), immune and inflammatory responses (such as inflammatory response, TNF-α signaling via NF-κB, and B cell receptor signaling), and osteogenic differentiation (such as BMP/TGF-β signaling and osteoblast differentiation) were curated from MSigDB and literature-derived sources. ssGSEA scores were compared across age groups using the Kruskal–Wallis test, with post-hoc pairwise comparisons performed using Dunn’s test with Bonferroni correction.

### 2.8. Immune Cell Infiltration Analysis

The CIBERSORT algorithm [28] was applied to estimate the relative proportions of 22 immune cell subtypes from bulk RNA-seq expression profiles using the LM22 signature matrix. For each sample, 1000 permutations were performed to ensure robustness, and only samples achieving a CIBERSORT *p* value < 0.05 were retained for downstream analysis. The resulting estimates of relative immune cell abundance were then compared across the three age groups using the Kruskal–Wallis test, with post-hoc pairwise comparisons conducted using Dunn’s test.

### 2.9. Development of the Pediatric-Specific Transcriptional Signature

To construct a pediatric-specific transcriptional signature, genes significantly upregulated in the pediatric group relative to both adolescents and adults were identified from the DEG analysis. Candidate genes were further refined based on their biological relevance to cell proliferation and metabolism, as determined by GO annotations, resulting in a final panel of 10 genes: MT1G, CTXN1, CENPV, CDCA7, HPDL, BNIP3, SIGMAR1, UCHL1, NPW, and TAC3. To quantify this pediatric-specific transcriptional signature at the individual level, a composite score was calculated for each sample as the mean of the z-score–transformed expression values of these 10 genes. Patients were subsequently stratified into high- and low-score groups using the median signature score as the cutoff.

### 2.10. Statistical Analysis

Continuous variables were summarized as median with interquartile range (IQR) and compared using the Kruskal–Wallis test for multi-group comparisons or the Mann–Whitney U test for two-group comparisons, whereas categorical variables were expressed as frequencies (percentages) and analyzed using the chi-square test or Fisher’s exact test, as appropriate. For survival analysis, overall survival (OS) and progression-free survival (PFS) were estimated using the Kaplan–Meier method, with differences between groups assessed by the log-rank test; hazard ratios (HRs) and corresponding 95% confidence intervals (CIs) were derived from Cox proportional hazards regression models. All statistical analyses were conducted using R (v4.2.0), with a two-sided *p* value < 0.05 considered statistically significant throughout.

## 3. Results

### 3.1. Patient Cohort and Baseline Clinicopathologic Characteristics

A total of 70 patients with pathologically confirmed primary osteosarcoma were enrolled in this study (Table 1, Figure 1). The median age at diagnosis was 14.0 years (interquartile range [IQR], 11.2–16.0 years), consistent with the well-established peak incidence of osteosarcoma in children and adolescents, supporting the representativeness of the present cohort. Males accounted for 54.3% (38/70) of all cases. Primary tumor sites were predominantly located in the femur (62.9%) and tibia (18.6%), followed by the humerus (17.1%), reflecting the characteristic anatomical predilection of osteosarcoma for the long bones of the extremities. The overwhelming majority of cases were classified as conventional osteosarcoma (68/70, 97.1%), with only a small minority representing other histologic subtypes, including one telangiectatic osteosarcoma and one parosteal osteosarcoma. With respect to treatment, all patients with available therapeutic data received standard neoadjuvant chemotherapy followed by surgical resection; subsequently, 15 patients (21.7%) received targeted therapy and 17 (24.6%) received immunotherapy based on postoperative pathologic and clinical assessments. Tumor tissue specimens obtained via biopsy or surgical resection were subjected to transcriptomic profiling by RNA sequencing. The median follow-up duration was 22.1 months (IQR, 17.0–28.5 months).

To delineate the molecular boundaries across the age continuum, the study population was systematically categorized into three developmental stages: pediatric (≤14 years; *n* = 37), adolescent (15–18 years; *n* = 22), and adult (≥19 years; *n* = 11) [19,20]. While chronological age expectedly diverged across these strata (Kruskal–Wallis test, *p* < 0.001), the subgroups exhibited a high degree of phenotypic homogeneity regarding their core clinicopathologic architecture. Specifically, no statistically significant disparities were observed in sex distribution, primary tumor location, or treatment history, including the use of neo-adjuvant chemotherapy and postoperative targeted/immunotherapy (all *p* > 0.05; Table 1). Interestingly, both non-conventional subtypes (telangiectatic and parosteal) were identified exclusively within the adolescent group (15–18 years). This suggests a higher degree of histological heterogeneity within the adolescent population compared to the pediatric and adult groups. However, no statistically significant difference was found in the overall distribution of major pathological types among the three groups (*p* > 0.05). This achieved statistical parity among baseline covariates is crucial, as it effectively decouples chronological age from potential confounding clinical factors, thereby establishing a rigorous analytical framework for isolating age-specific transcriptomic signatures.

### 3.2. Age-Associated Transcriptomic Heterogeneity in Osteosarcoma

**Figure 2:**
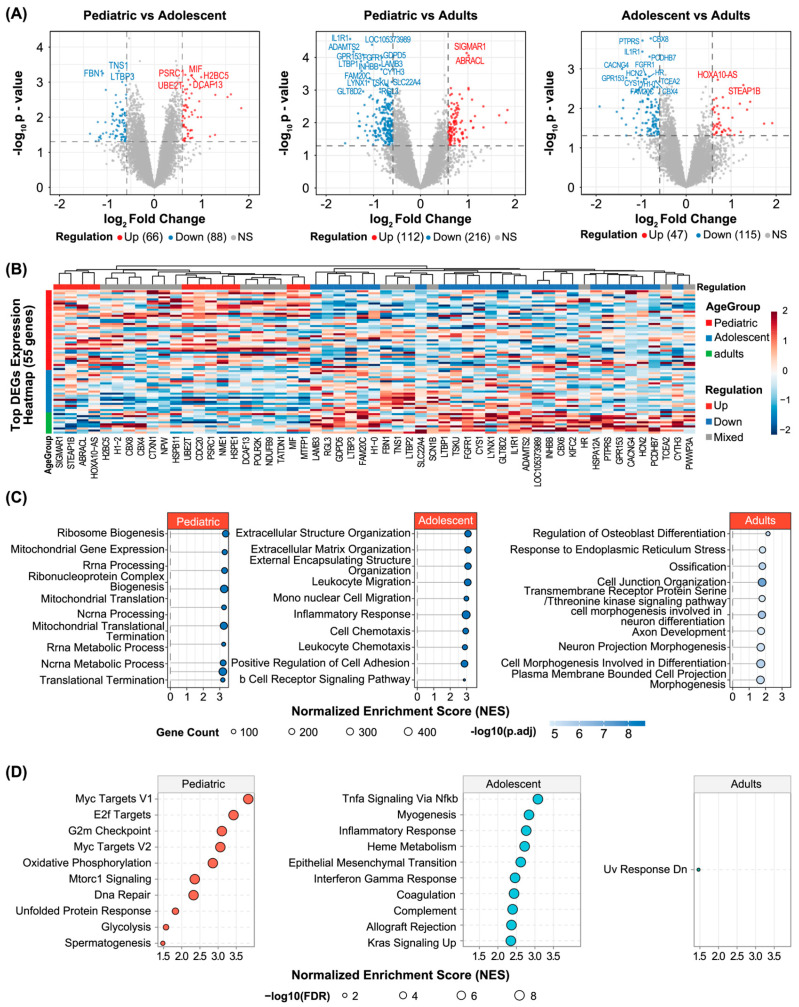
Age-stratified transcriptomic landscape reveals distinct molecular phenotypes across pediatric, adolescent, and adult osteosarcoma. (**A**) Volcano plots showing differentially expressed genes (DEGs) for the comparisons of Pediatric vs. Adolescent (left) and Pediatric vs. Adults (right). Red dots indicate significantly upregulated genes, while blue dots indicate downregulated genes, defined by |log_2_FC| > 0.58 and adjusted *p* < 0.05. (**B**) Hierarchical clustering heatmap of the top 55 differentially expressed genes (DEGs) across all samples. Each column represents a patient sample, and rows represent individual genes, color-coded by Z-score normalized expression. (**C**) Gene Ontology (GO) enrichment analysis showing the top 10 biological processes significantly enriched in the pediatric group, with the *X*-axis representing the −log_10_(*p*-value). (**D**) Gene Set Enrichment Analysis (GSEA) results highlighting distinct hallmark pathways. The top panels show hyperproliferative pathways (e.g., E2F targets) in pediatric cases, while the lower panels show immune and differentiation-related pathways enriched in adolescent and adult groups.

To delineate age-related transcriptional differences, pairwise differential gene expression analyses were performed among the three age groups. Comparison between pediatric patients and adults revealed 328 significantly differentially expressed genes (DEGs) (Figure 2A). In contrast, markedly fewer DEGs were identified between pediatric patients and adolescents (154 DEGs) and between adolescents and adults (162 DEGs) (Figure 2A), indicating that transcriptomic profiles are more convergent between chronologically proximate age groups. Unsupervised hierarchical clustering based on age-associated DEGs demonstrated that pediatric samples formed a relatively cohesive cluster, whereas adolescent samples occupied an intermediate position between the pediatric and adult clusters, with partial overlap with either group (Figure 2B). This pattern highlights a “transitional” transcriptomic phenotype along the age continuum rather than completely discrete molecular entities.

**Figure 3:**
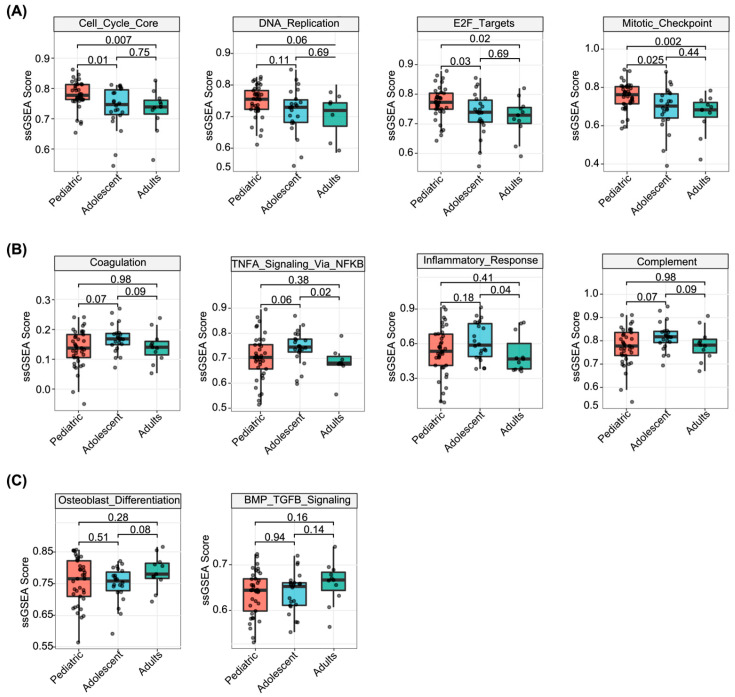
Single-sample gene set enrichment analysis (ssGSEA) reveals age-dependent activation of proliferative, immune, and osteogenic differentiation programs. (**A**) Boxplots comparing ssGSEA scores for cell cycle-related pathways (Cell Cycle Core, DNA Replication, E2F Targets) across the three age groups. The pediatric group shows significantly higher enrichment of these proliferation markers (*p* < 0.05). (**B**) Comparison of enrichment scores for differentiation and metabolic pathways, including Coagulation and Osteoblast Differentiation. (**C**) Comparison of signaling pathways, including TNF-α signaling via NF-κB and BMP/TGF-β signaling. Each dot represents an individual sample; *p*-values were calculated using the Kruskal–Wallis test.

Functional annotation of the DEGs revealed distinct biological programs enriched in each age stratum. In the pediatric group, genes associated with cell cycle progression and proliferation—including CDKN2A and CCNE1—were significantly upregulated (Figure 2A). Correspondingly, Gene Set Enrichment Analysis (GSEA) of Gene Ontology (GO) terms and Hallmark pathways demonstrated that the pediatric group was markedly enriched for translational termination, E2F targets, and G2M checkpoint pathways (Figure 2C,D), indicative of heightened proliferative activity and active anabolic metabolism. Single-sample GSEA (ssGSEA) further quantified pathway-level activation, confirming that pediatric samples exhibited the highest enrichment scores for mitotic checkpoint, cell cycle core, DNA replication, and E2F target gene sets, followed by adolescents and adults (Figure 3A). Collectively, these findings establish a hyperproliferative and metabolically active transcriptional signature as a hallmark of pediatric osteosarcoma.

Adolescent osteosarcoma was characterized by prominent inflammatory and immune-related transcriptional features. Differential expression analysis identified significant upregulation of immune-associated genes such as CCL4, CCL5, and IL32 in the adolescent group. GO and Hallmark enrichment analyses revealed activation of B cell receptor signaling, inflammatory response, and TNF-α signaling via NF-κB pathways (Figure 2C,D). ssGSEA corroborated these findings, demonstrating that adolescents exhibited the highest scores across immune- and inflammation-related gene sets compared with pediatric and adult groups (Figure 3B). These observations suggest that adolescent osteosarcoma represents a biologically distinct phase at which immune and inflammatory programs are most prominently engaged.

Adult osteosarcoma exhibited a transcriptional profile characterized by preferential activation of osteogenic differentiation programs. GO analysis revealed that genes specifically upregulated in adults were enriched in osteoblast differentiation and bone matrix remodeling processes (Figure 2C). At the gene set level, ssGSEA demonstrated that adults had the highest pathway activity scores for osteoblast differentiation and BMP/TGF-β signaling, whereas pediatric and adolescent groups showed comparatively lower enrichment (Figure 3C). Taken together, these results delineate an age-dependent transcriptomic trajectory: from a hyperproliferative “pediatric-type” phenotype, through an immune/inflammatory-activated adolescent intermediate state, toward an osteogenic differentiation-oriented adult phenotype.

### 3.3. Age-Dependent Differences in the Tumor Immune Microenvironment

**Figure 4:**
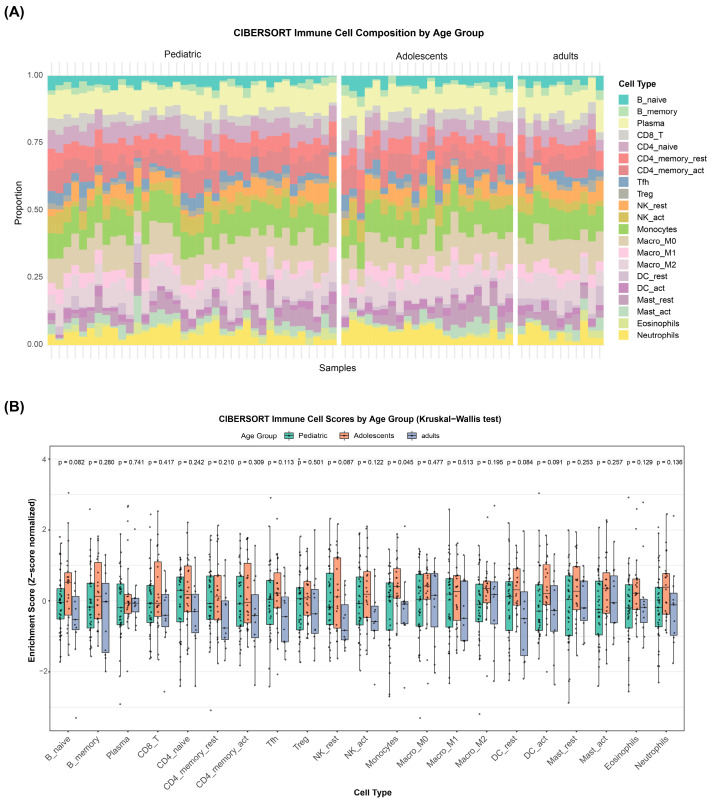
CIBERSORT-inferred immune cell composition reveals heightened immune activity in adolescent osteosarcoma. (**A**) Stacked bar plot illustrating the relative proportions of 22 infiltrating immune cell types in each patient sample, categorized by age group. (**B**) Detailed comparison of specific immune cell infiltration scores between pediatric, adolescent, and adult patients. *p*-values are indicated above each plot. Abbreviations for immune cells: B_naive, naïve B cells; B_memory, memory B cells; Plasma, plasma cells; CD8_T, CD8^+^ T cells; CD4_naive, naïve CD4^+^ T cells; CD4_memory_rest, resting memory CD4^+^ T cells; CD4_memory_act, activated memory CD4^+^ T cells; Tfh, T follicular helper cells; Treg, regulatory T cells; NK_rest, resting natural killer cells; NK_act, activated natural killer cells; Monocytes, monocytes; Macro M0/M1/M2, M0/M1/M2 macrophages; DC_rest/act, resting/activated dendritic cells; Mast_rest/act, resting/activated mast cells; Eosinophils, eosinophils; Neutrophils, neutrophils.

To further characterize the immune landscape across age groups, we applied the CIBERSORT algorithm to deconvolve the transcriptomic data and estimate immune cell infiltration. A complex immune infiltrate comprising both innate and adaptive immune cell populations was detected across all three age groups, including B cells (naïve, memory, and plasma cells), multiple T cell subsets (CD8^+^ T cells, naïve CD4^+^ T cells), natural killer (NK) cells, monocytes/macrophages (M0, M1, M2 polarization states), dendritic cells (resting and activated), mast cells (resting and activated), eosinophils, and neutrophils (Figure 4A).

Quantitative comparison of the relative abundance of 22 immune cell subtypes across age groups revealed that the adolescent group harbored a significantly higher proportion of monocytes than the pediatric and adult groups (Kruskal–Wallis *p* = 0.05; Figure 4B). Additionally, naïve B cells, resting NK cells, and resting dendritic cells showed trends toward higher enrichment in the adolescent group (0.05 < *p* < 0.1). These immune cell composition data further corroborate the observation that the adolescent osteosarcoma microenvironment is characterized by heightened immune–inflammatory activity relative to the pediatric and adult groups, consistent with the transcriptome-level pathway activation patterns described above.

### 3.4. A Pediatric-Specific 10-Gene Transcriptional Signature Stratifies Prognosis

**Figure 5:**
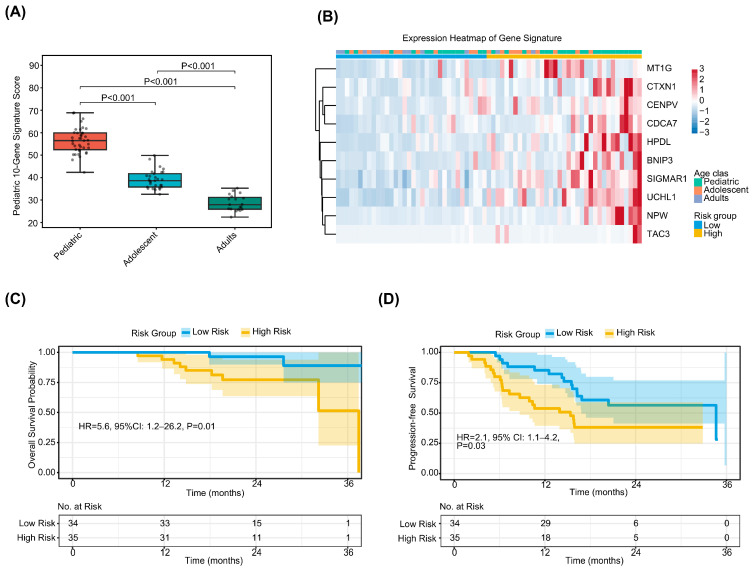
A pediatric-specific 10-gene transcriptional signature stratifies prognosis in the PKUPH-OS cohort. (**A**) Distribution of risk scores calculated by the 10-gene signature across the age groups. Pediatric patients exhibit significantly higher risk scores compared to adolescent and adult patients (*p* < 0.001). (**B**) Heatmap showing the expression of the 10 signature genes (MT1G, CTXN1, CENPV, CDCA7, UCHL1, HPDL, BNIP3, SIGMAR1, NPW, TAC3) in high-risk vs. low-risk groups. (**C**) Kaplan–Meier curve for overall survival (OS) in the discovery cohort. Patients are stratified into high-risk and low-risk groups based on the median risk score. Hazard ratio (HR) and log-rank *p*-value are shown. (**D**) Kaplan–Meier curve for progression-free survival (PFS) in the discovery cohort, demonstrating the prognostic utility of the signature in predicting tumor progression.

Building on the observation that pediatric osteosarcoma exhibits a distinct hyperproliferative transcriptomic phenotype, we sought to develop and validate a pediatric-specific transcriptional signature with prognostic utility. A panel of 10 genes associated with cell proliferation and metabolism—MT1G, CTXN1, CENPV, CDCA7, HPDL, BNIP3, SIGMAR1, UCHL1, NPW, and TAC3—was derived from genes significantly upregulated in the pediatric group relative to adolescents and adults. A composite gene set score was calculated for each sample. As anticipated, this score demonstrated a significant stepwise decline with increasing age (Figure 5A), confirming its validity as a molecular surrogate for the pediatric transcriptional phenotype.

Patients were stratified into high- and low-score groups based on the median signature score (*n* = 35 each; Figure 5B). The high-score group was significantly younger at diagnosis compared with the low-score group (median age 13.0 [IQR 9.5–15.0] vs. 15.0 [IQR 13.0–17.0] years; *p* = 0.01; Table 2). No significant differences were observed between groups with respect to other baseline clinicopathologic characteristics, including sex, primary tumor site, histologic subtype, use of chemotherapy, targeted therapy, immunotherapy, and tumor necrosis rate (all *p* > 0.05). This indicates that the 10-gene signature captures a biological dimension that is only partially overlapping with chronological age and is largely independent of conventional clinical factors.

Survival analysis demonstrated marked prognostic discrimination by the 10-gene signature. After a median follow-up of 22.1 months, Kaplan–Meier analyses revealed that the high-score group experienced significantly inferior overall survival (OS) (unadjusted HR = 5.6, 95% CI: 1.2–26.2; log-rank *p* = 0.01; Figure 5C) and progression-free survival (PFS) (unadjusted HR = 2.1, 95% CI: 1.1–4.2; log-rank *p* = 0.03; Figure 5D) compared with the low-score group. These results indicate that a transcriptional program characteristic of pediatric tumors is paradoxically associated with adverse prognosis within a clinically heterogeneous osteosarcoma cohort.

**Figure 6:**
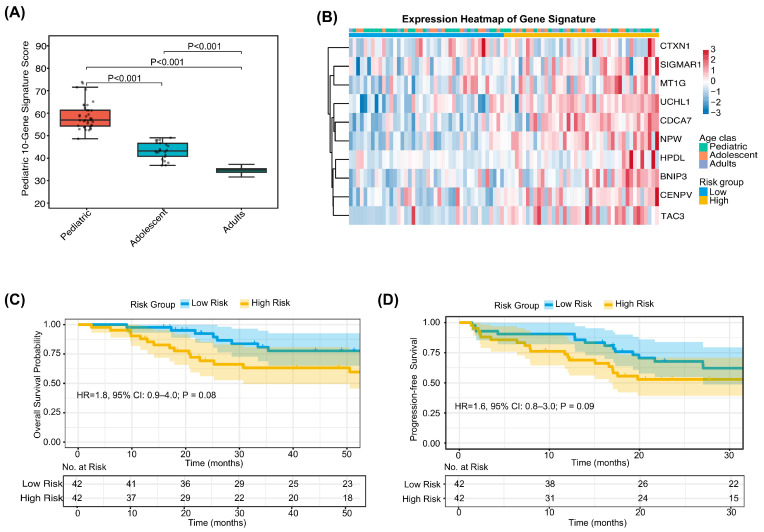
External validation of the pediatric-specific 10-gene transcriptional signature in the TARGET-OS cohort. (**A**) Boxplot showing the validation of the 10-gene risk score distribution in the independent cohort, confirming the high-risk profile associated with pediatric status. (**B**) Heatmap of signature gene expression in the validation cohort, demonstrating consistent expression patterns with the discovery set. (**C**) Kaplan–Meier analysis of overall survival (OS) in the validation cohort. (**D**) Kaplan–Meier analysis of progression-free survival (PFS) in the validation cohort.

To assess the external validity and generalizability of the pediatric transcriptional signature, we evaluated its performance in the independent TARGET-OS cohort, which comprises transcriptomic data from 87 patients with primary osteosarcoma. Consistent with findings in the discovery cohort, the signature score in the TARGET-OS cohort exhibited a significant stepwise decline with increasing age (Figure 6A), reinforcing its reliability as a marker of the pediatric osteosarcoma phenotype. Upon stratification by the median score (Figure 6B), Kaplan–Meier analysis revealed that the high-score group demonstrated inferior OS (unadjusted HR = 1.8, 95% CI: 0.9–4.0; log-rank *p* = 0.08) and PFS (unadjusted HR = 1.6, 95% CI: 0.8–3.0; log-rank *p* = 0.09; Figure 6C,D). Although borderline statistical significance was observed—likely attributable to sample size limitations—the direction and magnitude of the effect were concordant with those in the PKUPH-OS cohort, supporting the cross-cohort reproducibility and potential clinical utility of this pediatric-specific transcriptional signature.

## 4. Discussion

The present study provides a systematic transcriptomic characterization of osteosarcoma across three distinct age strata, addressing a critical gap in our understanding of the molecular heterogeneity underlying this malignancy’s striking bimodal clinical distribution. By analyzing 70 clinical specimens stratified into pediatric (≤14 years), adolescent (15–18 years), and adult (≥19 years) cohorts, we identified three biologically distinct molecular phenotypes: a hyperproliferative program in pediatric cases, a unique transition toward an immune–inflammatory signature in adolescents, and a shift toward osteogenic differentiation pathways in adults. Furthermore, our 10-gene prognostic signature demonstrated a stepwise expression decline with increasing age and was associated with inferior prognosis across cohorts, highlighting its potential as a clinically actionable tool for risk stratification. These findings reinforce the notion that chronological age is not merely a demographic variable but a fundamental determinant of the osteosarcoma transcriptomic landscape, necessitating age-adapted strategies in both biological research and precision clinical management.

A central finding of our study is that pediatric osteosarcoma exhibits extreme hyperproliferation coupled with relative immune suppression, displaying the highest enrichment for E2F targets, G2M checkpoint, and DNA replication pathways, while showing lower immune activation signatures than adolescent counterparts. We posit that this molecular phenotype is intrinsically linked to the physiological context of the pediatric skeleton, specifically the peak activity of the epiphyseal growth plate during late childhood. The intense longitudinal bone growth and rapid cell turnover characteristic of this developmental stage likely act as a catalyst for the accumulation of the genomic aberrations we observed, such as MYC and CCNE1 amplifications and homologous recombination deficiency (HRD) [7,11]. Our findings align with the paradigm that MYC-driven metabolic competition and CCNE1-induced replication stress not only fuel cellular proliferation [29] but also promote an immunologically “cold” microenvironment through the suppression of antigen presentation and the upregulation of inhibitory signals like PD-L1 [30,31]. Multiple studies have characterized osteosarcoma as immunologically “cold” with M2-polarized macrophages and limited T cell infiltration, with high copy number variation (CNV)/genomic instability tumors—features enriched in pediatric cases—exhibiting the most immunosuppressive microenvironments [14,17,32,33]. From a clinical perspective, this hyperproliferative program, coupled with an HRD background, provides a strong biological rationale for exploring therapeutic combinations of PARP inhibitors and cell-cycle checkpoint inhibitors specifically in pediatric cohorts [34,35]. Furthermore, the enrichment of VEG-FA-related signaling in these cases suggests that anti-angiogenic agents could be leveraged to potentially convert this immunosuppressive “cold” microenvironment [36,37], offering a more tailored precision strategy for children with aggressive disease.

In contrast to pediatric tumors, adolescent osteosarcoma displayed a distinct immune–inflammatory transcriptional profile. Our analyses revealed that adolescent tumors exhibited maximal enrichment for inflammatory response, TNFα–NF-κB signaling, and B cell receptor pathways, accompanied by significant upregulation of immune-related genes including CCL4, CCL5, and IL32. This “intermediate” molecular phenotype appears to be shaped by the unique physiological milieu of the peri-pubertal period. The systemic surge in growth hormone (GH) and insulin-like growth factor 1 (IGF-1), alongside rising levels of sex hormones (estrogen and testosterone) during adolescence [38], establishes a dynamic environment that likely modulates the intratumoral immune landscape [39,40]. Accumulating evidence indicates that the GH/IGF-1 axis can enhance the production of pro-inflammatory cytokines, while sex hormones are known to influence the maturation of the immune system [41]. These physiological factors align with the significant enrichment of monocyte and B cell signatures observed in the adolescent cohort in this study. Furthermore, the transition from rapid longitudinal growth to bone remodeling and consolidation during late adolescence may provide a more “permissive” microenvironment for immune cell infiltration, contrasting with the extreme hyperproliferative state that dominates the pediatric skeleton. Consequently, adolescent osteosarcoma occupies a distinct biological window where developmental and hormonal cues facilitate heightened immune engagement. From a therapeutic perspective, this relatively elevated immune activity provides a rationale for exploring immunotherapy or immunochemotherapy combinations specifically in the adolescent population, as this group may exhibit differential responsiveness compared to the immunologically “colder” pediatric subgroup.

The pediatric-specific 10-gene transcriptional signature (MT1G, CTXN1, CENPV, CDCA7, HPDL, BNIP3, SIGMAR1, UCHL1, NPW, TAC3) captures a program enriched for cell proliferation and metabolic reprogramming, with constituent genes linked to MYC-driven transcription (CDCA7), chromosome segregation (CENPV) [42], and hypoxia-responsive autophagy (BNIP3) [43]. Notably, while the signature score decreases stepwise with advancing age—consistent with its derivation from pediatric-upregulated genes—higher scores predict significantly inferior overall survival and progression-free survival, indicating that this hyperproliferative program confers aggressive tumor biology across all age groups. This finding resonates with reports linking MYC amplification and HRD phenotypes in pediatric osteosarcoma to poor prognosis [30,44]. External validation in the TARGET-OS cohort demonstrated the expected age-dependent score decline and showed concordant trends toward inferior survival outcomes; although formal statistical significance was not achieved—likely due to sample size limitations—the consistent effect direction across independent datasets supports reproducibility. Consequently, the signature currently serves as a hypothesis-generating tool rather than a definitive clinical classifier. Its potential clinical utility may lie in its future integration into multi-dimensional risk models that combine transcriptomic data with genomic and epigenomic features. Such models could eventually assist in identifying high-risk candidates for treatment intensification or for early-phase trials of targeted agents addressing MYC-driven biology or homologous recombination deficiency (HRD) [31,45].

Several limitations of this study merit acknowledgment. First, although the total cohort size is representative of this rare pathology, the sample size, particularly for the adult subgroup (*n* = 11), remains limited. Consequently, the findings in the adult group should be interpreted as preliminary and require validation in larger, multi-institutional cohorts. Second, while the 10-gene signature demonstrated a consistent directional trend in the external TARGET-OS cohort, it did not achieve formal statistical significance. As previously discussed, this likely stems from differences in cohort composition and sample size, reinforcing the need for broader prospective validation. Third, the current study is primarily descriptive and cross-sectional in nature. While we have provided biological rationales based on hormonal and developmental contexts, we lack direct functional validation through patient-derived organoids or xenograft models. Future research utilizing targeted gene perturbation is necessary to establish causal relationships between the identified signatures and aggressive phenotypes.

## 5. Conclusions

This study provides a comprehensive transcriptomic framework demonstrating that pediatric, adolescent, and adult osteosarcoma represent biologically distinct disease entities rather than a uniform malignancy. We identified age-specific molecular phenotypes—hyperproliferative programs in pediatric tumors, immune–inflammatory activation in adolescent tumors, and osteogenic differentiation in adult tumors—that may underlie the recognized clinical heterogeneity across age groups. The pediatric-specific 10-gene signature showed significant prognostic associations in the discovery cohort and concordant trends in external validation, though further confirmation in larger cohorts is warranted before clinical application. These findings support the concept of age-adapted therapeutic strategies and provide a foundation for developing more refined, biologically informed risk stratification approaches in osteosarcoma.

## Figures and Tables

**Table 1:** Baseline clinicopathologic characteristics of the PKUPH-OS cohort and comparison among age groups.

Characteristic	All Patients	Pediatric (≤14 y)	Adolescent (15–18 y)	Adult (≥19 y)	*p* Value
No. of patients	70	37	22	11	
Age, years, median (IQR)	14.0 (11.2–16.0)	12.0 (9.0–13.0)	15.5 (15.0–16.0)	24.0 (20.5–32.0)	<0.001
Sex, *n* (%)					0.76
Male	38 (54.3%)	20 (54.1%)	13 (59.1%)	5 (45.5%)	
Female	32 (45.7%)	17 (45.9%)	9 (40.9%)	6 (54.5%)	
Primary tumor site, *n* (%)					0.92
Femur	44 (62.9%)	24 (64.9%)	14 (63.6%)	6 (54.5%)	
Tibia	13 (18.6%)	7 (18.9%)	4 (18.2%)	2 (18.2%)	
Humerus	12 (17.1%)	5 (13.5%)	4 (18.2%)	3 (27.3%)	
Fibula	1 (1.4%)	1 (2.7%)	0 (0.0%)	0 (0.0%)	
Histologic subtype, *n* (%)					0.11
Conventional osteosarcoma	68 (97.1%)	37 (100.0%)	20 (90.9%)	11 (100.0%)	
Other subtypes	2 (2.9%)	0 (0.0%)	2 (9.1%)	0 (0.0%)	
Chemotherapy, *n* (%)					—
Yes	70 (100.0%)	37 (100.0%)	22 (100.0%)	11 (100.0%)	
No	0 (0.0%)	0 (0.0%)	0 (0.0%)	0 (0.0%)	
Tumor necrosis rate (%), median (IQR)	82.5 (56.8–95.2)	80.2 (63.9–94.9)	88.2 (62.6–96.0)	79.8 (6.5–93.9)	0.89
Targeted therapy, *n* (%)					0.87
Yes	15 (21.7%)	8 (21.6%)	4 (19.0%)	3 (27.3%)	
No	54 (78.3%)	29 (78.4%)	17 (81.0%)	8 (72.7%)	
Immunotherapy, *n* (%)					0.98
Yes	17 (24.6%)	9 (24.3%)	5 (23.8%)	3 (27.3%)	
No	52 (75.4%)	28 (75.7%)	16 (76.2%)	8 (72.7%)	

Continuous variables are presented as median (IQR) and were compared using the Kruskal–Wallis test. Categorical variables are presented as *n* (%) and were compared using the χ^2^ test. Percentages are calculated among patients with available data (missing values excluded).

**Table 2:** Association between pediatric gene-set risk score (high vs. low) and clinicopathologic characteristics in the PKUPH-OS cohort.

Variable	High-Risk Group (*n* = 35)	Low-Risk Group (*n* = 35)	*p*-Value
Age, years, median (IQR)	13.0 (9.5–15.0)	15.0 (13.0–17.0)	0.01
Age group, *n* (%)			0.08
Pediatric (≤14 y)	23 (65.7%)	14 (40.0%)	
Adolescent (15–18 y)	9 (25.7%)	13 (37.1%)	
Adult (≥19 y)	3 (8.6%)	8 (22.9%)	
Sex, *n* (%)			0.47
Male	21 (60.0%)	17 (48.6%)	
Female	14 (40.0%)	18 (51.4%)	
Primary site, *n* (%)			0.08
Femur	18 (51.4%)	26 (74.3%)	
Others	17 (48.6%)	9 (25.7%)	
Pathologic subtype, *n* (%)			1.00
Conventional	34 (97.1%)	34 (97.1%)	
Others	1 (2.9%)	1 (2.9%)	
Chemotherapy, *n* (%)			1.00
No	0 (0.0%)	0 (0.0%)	
Yes	35 (100.0%)	34 (100.0%)	
Targeted therapy, *n* (%)			1.00
No	27 (77.1%)	27 (79.4%)	
Yes	8 (22.9%)	7 (20.6%)	
Immunotherapy, *n* (%)			1.00
No	26 (74.3%)	26 (76.5%)	
Yes	9 (25.7%)	8 (23.5%)	
Tumor necrosis rate (%), median (IQR)	82.9 (51.3–96.6)	71.4 (62.6–92.4)	0.44

## Data Availability

The sequencing data reported in this study have been deposited in the Gene Expression Omnibus (GEO) database of the National Center for Biotechnology Information (NCBI) under accession number GSE317512 and are publicly accessible at https://www.ncbi.nlm.nih.gov/geo/ (accessed on 26 January 2026). The TARGET-OS dataset is publicly available at https://ocg.cancer.gov/programs/target (accessed on 23 October 2024).

## References

[1] Mirabello L., Troisi R.J., Savage S.A. (2009). Osteosarcoma Incidence and Survival Rates from 1973 to 2004. Cancer.

[2] Ottaviani G., Jaffe N. (2009). Pediatric and Adolescent Osteosarcoma. Cancer Treatment and Research.

[3] Broadhead M.L., Clark J.C.M., Myers D.E., Dass C.R., Choong P.F.M. (2011). The Molecular Pathogenesis of Osteosarcoma: A Review. Sarcoma.

[4] Isakoff M.S., Bielack S.S., Meltzer P., Gorlick R. (2015). Osteosarcoma: Current Treatment and a Collaborative Pathway to Success. J. Clin. Oncol..

[5] Gill J., Gorlick R. (2021). Advancing Therapy for Osteosarcoma. Nat. Rev. Clin. Oncol..

[6] Bielack S.S., Hecker-Nolting S., Blattmann C., Kager L. (2016). Advances in the Management of Osteosarcoma. F1000Research.

[7] Chen X., Bahrami A., Pappo A., Easton J., Dalton J., Hedlund E., Ellison D., Shurtleff S., Wu G., Wei L. (2014). Recurrent Somatic Structural Variations Contribute to Tumorigenesis in Pediatric Osteosarcoma. Cell Rep..

[8] Behjati S., Tarpey P.S., Haase K., Ye H., Young M.D., Alexandrov L.B., Farndon S.J., Collord G., Wedge D.C., Martincorena I. (2017). Recurrent Mutation of IGF Signalling Genes and Distinct Patterns of Genomic Rearrangement in Osteosarcoma. Nat. Commun..

[9] Kim C., Davis L.E., Albert C.M., Samuels B., Roberts J.L., Wagner M.J. (2023). Osteosarcoma in Pediatric and Adult Populations: Are Adults Just Big Kids?. Cancers.

[10] Gianferante D.M., Mirabello L., Savage S.A. (2017). Germline and Somatic Genetics of Osteosarcoma—Connecting Aetiology, Biology and Therapy. Nat. Rev. Endocrinol..

[11] Zou C., Huang R., Lin T., Wang Y., Tu J., Zhang L., Wang B., Huang J., Zhao Z., Xie X. (2024). Age-Dependent Molecular Variations in Osteosarcoma: Implications for Precision Oncology across Pediatric, Adolescent, and Adult Patients. Front. Oncol..

[12] Tang S., Roberts R.D., Cheng L., Li L. (2023). Osteosarcoma Multi-Omics Landscape and Subtypes. Cancers.

[13] Outani H., Ikegami M., Imura Y., Nakai S., Takami H., Kotani Y., Inoue A., Okada S. (2025). Age-Related Genomic Alterations and Chemotherapy Sensitivity in Osteosarcoma: Insights from Cancer Genome Profiling Analyses. Int. J. Clin. Oncol..

[14] Zhou Y., Yang D., Yang Q., Lv X., Huang W., Zhou Z., Wang Y., Zhang Z., Yuan T., Ding X. (2020). Single-Cell RNA Landscape of Intratumoral Heterogeneity and Immunosuppressive Microenvironment in Advanced Osteosarcoma. Nat. Commun..

[15] Liu Y., Feng W., Dai Y., Bao M., Yuan Z., He M., Qin Z., Liao S., He J., Huang Q. (2021). Single-Cell Transcriptomics Reveals the Complexity of the Tumor Microenvironment of Treatment-Naive Osteosarcoma. Front. Oncol..

[16] Zheng X., Liu X., Zhang X., Zhao Z., Wu W., Yu S. (2024). A Single-Cell and Spatially Resolved Atlas of Human Osteosarcomas. J. Hematol. Oncol..

[17] Liu W., Hu H., Shao Z., Lv X., Zhang Z., Deng X., Song Q., Han Y., Guo T., Xiong L. (2023). Characterizing the Tumor Microenvironment at the Single-Cell Level Reveals a Novel Immune Evasion Mechanism in Osteosarcoma. Bone Res..

[18] Yang Y., Huang Z., Yuan M., Rui J., Chen R., Jin T., Sun Y., Deng Z., Shan H., Niu X. (2023). Genomic and Transcriptomic Characterization of Pre-Operative Chemotherapy Response in Patients with Osteosarcoma. Sci. Rep..

[19] Testa S., Hu B.D., Saadeh N.L., Pribnow A., Spunt S.L., Charville G.W., Bui N.Q., Ganjoo K.N. (2021). A Retrospective Comparative Analysis of Outcomes and Prognostic Factors in Adult and Pediatric Patients with Osteosarcoma. Curr. Oncol..

[20] Goulding D., Arguinchona L., Anderson-Mellies A., Mikkelsen M., Eguchi M., Marinoff H., Zahedi S., Ribeiro K.B., Cockburn M., Galindo C.R. (2023). Sociodemographic Disparities in Presentation and Survival of Pediatric Bone Cancers. J. Pediatr. Hematol. Oncol..

[21] Dobin A., Davis C.A., Schlesinger F., Drenkow J., Zaleski C., Jha S., Batut P., Chaisson M., Gingeras T.R. (2012). STAR: Ultrafast Universal RNA-Seq Aligner. Bioinformatics.

[22] Liao Y., Smyth G.K., Shi W. (2014). featureCounts: An Efficient General Purpose Program for Assigning Sequence Reads to Genomic Features. Bioinformatics.

[23] Love M.I., Huber W., Anders S. (2014). Moderated Estimation of Fold Change and Dispersion for RNA-Seq Data with DESeq2. Genome Biol..

[24] Gu Z., Eils R., Schlesner M. (2016). Complex Heatmaps Reveal Patterns and Correlations in Multidimensional Genomic Data. Bioinformatics.

[25] Wu T., Hu E., Xu S., Chen M., Guo P., Dai Z., Feng T., Zhou L., Tang W., Zhan L. (2021). clusterProfiler 4.0: A Universal Enrichment Tool for Interpreting Omics Data. Innov..

[26] Liberzon A., Birger C., Thorvaldsdóttir H., Ghandi M., Mesirov J.P., Tamayo P. (2015). The Molecular Signatures Database Hallmark Gene Set Collection. Cell Syst..

[27] Hänzelmann S., Castelo R., Guinney J. (2013). GSVA: Gene Set Variation Analysis for Microarray and RNA-Seq Data. BMC Bioinform..

[28] Newman A.M., Liu C.L., Green M.R., Gentles A.J., Feng W., Xu Y., Hoang C.D., Diehn M., Alizadeh A.A. (2015). Robust Enumeration of Cell Subsets from Tissue Expression Profiles. Nat. Methods.

[29] Nagy M.R., Puopolo O., Alston E., Challa S., Ceca E., Li Y., Cherniack A.D., Lazo de la Vega L., Meyerson M., Church A.J. (2025). MYC Amplification and MYC Protein Expression Are Poor Prognostic Markers in Pediatric and Young Adult Osteosarcoma. Cancer.

[30] Noon S.D., Ijaz J., Coorens T.H., Amary F., Ye H., Strobl A., Lyskjær I., Flanagan A.M., Behjati S. (2021). MYC Amplifications Are Common Events in Childhood Osteosarcoma. J. Pathol. Clin. Res..

[31] Marinoff A.E., Spurr L.F., Fong C., Li Y.Y., Forrest S.J., Ward A., Doan D., Corson L., Mauguen A., Pinto N. (2023). Clinical Targeted Next-Generation Panel Sequencing Reveals MYC Amplification Is a Poor Prognostic Factor in Osteosarcoma. JCO Precis. Oncol..

[32] Wu C.-C., Beird H.C., Livingston J.A., Advani S., Mitra A., Cao S., Reuben A., Ingram D., Wang W.-L., Ju Z. (2020). Immuno-Genomic Landscape of Osteosarcoma. Nat. Commun..

[33] Tian H., Cao J., Li B., Nice E.C., Mao H., Zhang Y., Huang C. (2023). Managing the Immune Microenvironment of Osteosarcoma: The Outlook for Osteosarcoma Treatment. Bone Res..

[34] Keller K.M., Koetsier J., Schild L., Amo-Addae V., Eising S., Handel K.V.D., Ober K., Koopmans B., Essing A., van den Boogaard M.L. (2023). The Potential of PARP as a Therapeutic Target across Pediatric Solid Malignancies. BMC Cancer.

[35] Zoumpoulidou G., Alvarez-Mendoza C., Mancusi C., Ahmed R.-M., Denman M., Steele C.D., Tarabichi M., Roy E., Davies L.R., Manji J. (2021). Therapeutic Vulnerability to PARP1,2 Inhibition in RB1-Mutant Osteosarcoma. Nat. Commun..

[36] Voron T., Colussi O., Marcheteau E., Pernot S., Nizard M., Pointet A.-L., Latreche S., Bergaya S., Benhamouda N., Tanchot C. (2015). VEGF-A Modulates Expression of Inhibitory Checkpoints on CD8+ T Cells in Tumors. J. Exp. Med..

[37] Zhang C., Wang L., Xiong C., Zhao R., Liang H., Luo X. (2021). The Role of Vascular Endothelial Growth Factor as a Prognostic and Clinicopathological Marker in Osteosarcoma: A Systematic Review and Meta-Analysis. J. Orthop. Surg. Res..

[38] Dees W.L., Hiney J.K., Srivastava V.K. (2021). IGF-1 Influences Gonadotropin-Releasing Hormone Regulation of Puberty. Neuroendocrinology.

[39] Poudel S.B., Dixit M., Neginskaya M., Nagaraj K., Pavlov E., Werner H., Yakar S. (2020). Effects of GH/IGF on the Aging Mitochondria. Cells.

[40] Ucciferri C.C., Dunn S.E. (2022). Effect of Puberty on the Immune System: Relevance to Multiple Sclerosis. Front. Pediatr..

[41] Witkowska-Sędek E., Pyrżak B. (2020). Chronic Inflammation and the Growth Hormone/Insulin-like Growth Factor-1 Axis. Cent.-Eur. J. Immunol..

[42] Nabi D., Drechsler H., Pschirer J., Korn F., Schuler N., Diez S., Jessberger R., Chacón M. (2021). CENP-V Is Required for Proper Chromosome Segregation through Interaction with Spindle Microtubules in Mouse Oocytes. Nat. Commun..

[43] Azad M.B., Gibson S.B. (2010). Role of BNIP3 in Proliferation and Hypoxia-induced Autophagy: Implications for Personalized Cancer Therapies. Ann. N. Y. Acad. Sci..

[44] Jiang Y., Wang J., Sun M., Zuo D., Wang H., Shen J., Jiang W., Mu H., Ma X., Yin F. (2022). Multi-Omics Analysis Identifies Osteosarcoma Subtypes with Distinct Prognosis Indicating Stratified Treatment. Nat. Commun..

[45] Patel T.D., Grimm S.L., Kanchi R.S., Gandhi T., Koirala A., Yustein J.T., Coarfa C. (2024). Identification of an Early Survival Prognostic Gene Signature for Localized Osteosarcoma Patients. Sci. Rep..

